# Anti-Hyperuricemic, Anti-Arthritic, Hemolytic Activity and Therapeutic Safety of Glycoconjugated Triazole-Phthalimides

**DOI:** 10.3390/biomedicines11092537

**Published:** 2023-09-14

**Authors:** José Guedes da Silva, André de Lima Aires, Rebeca Xavier da Cunha, Talyta Valéria Siqueira do Monte, Shalom Pôrto de Oliveira Assis, Ronaldo Nascimento de Oliveira, Talita Giselly dos Santos Souza, Cristiano Aparecido Chagas, Jacinto da Costa Silva Neto, Hallysson Douglas Andrade de Araújo, Vera Lúcia de Menezes Lima

**Affiliations:** 1 Laboratório de Lipídeos e Aplicações de Biomoléculas em Doenças Prevalentes e Negligenciadas (LAB—DPN), Centro de Biociências, Departamento de Bioquímica, Universidade Federal de Pernambuco, Recife 50670-901, PE, Brazil; rebeca_xavier@live.com; 2 Faculdade de Medicina de Garanhuns (FAMEG), Garanhuns 55297-654, PE, Brazil; 3 Centro de Ciências Médicas—Área Acadêmica de Medicina Tropical, Universidade Federal de Pernambuco, Recife 50670-901, PE, Brazil; andre.laires@ufpe.br; 4 Centro de Ciências da Saúde (CCS), Departamento de Enfermagem, Universidade Federal de Pernambuco, Recife 50670-901, PE, Brazil; talyta.valeria@gmail.com; 5 Laboratório de Biotecnologia e Ciências Ambientais (NPCIAMB), Departamento de Medicina, Universidade Católica de Pernambuco (UNICAP), Recife 50050-900, PE, Brazil; shalom.porto@unicap.br; 6 Laboratório de Síntese de Compostos Bioativos (LSCB), Departamento de Química, Universidade Federal Rural de Pernambuco (UFRPE), Recife 52171-900, PE, Brazil; ronaldo.noliveira@ufrpe.br; 7 Laboratório de Biotecnologia e Fármacos, Centro Acadêmico de Vitória (CAV), Universidade Federal de Pernambuco (UFPE), Recife 50670-901, PE, Brazil; talitagiselly@hotmail.com (T.G.d.S.S.); cristiano.chagas@ufpe.br (C.A.C.); 8 Laboratório de Pesquisas Citológicas e Moleculares (LPCM), Universidade Federal de Pernambuco (UFPE), Recife 50670-901, PE, Brazil; jacintocosta@hotmail.com

**Keywords:** hyperuricemia, arthritis, medicinal chemistry, uric acid, gouty arthritis

## Abstract

Hyperuricemia, the metabolic alteration that leads to gout or gouty arthritis, is increasing worldwide. Glycoconjugated triazole-phthalimides show potent anti-inflammatory activity. The aim of this study was to evaluate the anti-hyperuricemia effect of glycoconjugated triazole-phthalimides. To develop hyperuricemia, groups of mice received orally potassium oxonate (250 mg/kg) for 7 days, and **F2**, **F3** and **F4** glycoconjugated triazole-phthalimides (20 mg/kg), allopurinol (300 mg/kg), and 1% carboxymethylcellulose; indomethacin (2 and 4 mg/kg) was the positive control for anti-arthritic effect. Genotoxic and mutagenic effects were evaluated by the comet and micronucleus assays, respectively. The hemolytic action of the compounds was evaluated. Phthalimides **F2**, **F3** and **F4** significantly reduced the levels of serum uric acid, creatinine and urea in hyperuricemic animals. In addition, the compounds were efficient in reducing protein denaturation in a dose-dependent manner. In an interesting way, the histopathological analysis of kidneys from groups treated with **F2**, **F3** and **F4** showed a glomerular architecture, with the Bowman’s capsule and renal tubules having a normal appearance and without inflammatory changes. Also, **F2** and **F4** showed a small increase in micronuclei, indicating a low mutagenic effect, whilst by comet assay only, we could infer that **F4** affected the frequency and damage index, thus indicating a very small genotoxic action. Similarly, the phthalimides showed a low degree of erythrocyte hemolysis (<3%). Our data demonstrate that the new glycoconjugate triazole-phthalimides have potential to treat hyperuricemia and its secondary complications, such as gouty arthritis, with a low to non-significant rate of erythrocytes hemolysis, genotoxicity and mutagenicity making these molecules strong candidates as pharmaceutical agents for treatment requiring uric-acid-lowering therapy.

## 1. Introduction

Uric acid is among the endogenous products synthetized by the liver, intestines, and the vascular endothelium. Uric acid arises because of the breakdown of purine molecules by the enzymatic action of xanthine oxidase. Purine is an aromatic heterocyclic compound formed by adenine and guanine found in nucleotides that serve as monomeric units of the nucleic acid [1]. Part of uric acid remains in the blood while the rest is eliminated by the kidneys or the gastrointestinal tract [2]. The levels of uric acid in the blood may increase due to increased endogenous production, difficulties in elimination, and interference with the use of certain medications [3,4], or even through the ingestion of foods rich in purines, such as animal protein, barley, soft drinks, distilled beverages, and high-fructose beverages [5].

When excess uric acid exceeds the solubility threshold, this leads to precipitation and the formation of small needle-shaped crystals. Serum monosodium urate above physiological levels (0.36 mmol/L or 6 mg/dL) is defined as hyperuricemia; these crystals are deposited in various places in the body [6]. The symptomatology is accompanied by a process of intense pain and local inflammation, with the joints of the lower limbs (knees, ankles, heels, toes) being the most affected [7].

Hyperuricemia has gained increasing prominence worldwide, since population aging corroborates with the increased rates and/or chances of individuals developing one of the most severe forms of this metabolic alteration, which is the pathogenesis of gout or gouty arthritis [8]. This scenario worries health authorities, especially in those countries that are going through a demographic transition with the characteristics of an aging population, with reports that men in adulthood are more affected than women [9,10]. Hyperuricemia can lead to complications such as heart failure, high blood pressure, kidney failure and diabetes mellitus [11]. In addition, several drugs used to treat other comorbidities can increase serum uric acid levels, such as diuretics, especially thiazides [12], immunosuppressants such as Ciclosporin [3], cytotoxic chemotherapy [13], and Levodopa [14], among other pharmaceutical formulations [15,16].

The most commonly used treatment for the diagnosis of gouty arthritis is therapy with non-steroidal anti-inflammatory drugs (NSAIDs), drugs prescribed to control pain, fever and inflammation [17]. However, the long-term use of NSAIDs can cause adverse renal and gastrointestinal effects [18]. Still, regarding this class of drugs, we can highlight corticosteroids and colchicine. Corticosteroids can cause weight gain, generating metabolic problems and fluid retention, compromising hemodynamic, cardiovascular, and renal functions [19], while colchicine can cause gastrointestinal complications and a reduction in circulating neutrophils, and high doses can cause aplastic anemia by inhibiting erythroid precursor mitosis and increase the risk of developing Alzheimer’s disease [20]. The most widespread hyperuricemia inhibitors are the xanthine oxidase inhibitorallopurinol and the uricosuric agent probenecid, which inhibit the reabsorption of urates at the level of the proximal convoluted tubule, and seeks to bring the serum uric acid level below 6 mg/dL (357 µmol/L). Both allopurinol and probenecid can cause adverse effects, such as leukopenia and flushing with the former drug and headache and irritation of the gastrointestinal tract with the latter drug [21]. The enzyme uricase extracted from the fungus Aspergillus flavus is used in the treatment of hyperuricemia. However, as it comes from a microorganism, the enzyme undergoes immune response reactions, decreasing its half-life and consequently the efficiency in catalyzing the breakdown of uric acid in the long term [22].

The therapy of hyperuricemia and the severity of gouty arthritis still present challenges, mainly due to adverse reactions and toxic effects of commercial therapeutic agents [23,24]. Therefore, the search for molecules that are efficient in reducing the serum level of uric acid in the hyperuricemic state and decreasing urates and glycoproteins and the inflammatory process of gouty arthritis is fundamental and urgent [25,26]. In this scenario, phthalimides are a group of cyclic imides with diverse biological activities, from hypolipidemic [27] to analgesic, antitumor, antimicrobial [28], antifungal [29], antiparasitic [30], antiviral for the treatment and/or prophylaxis of COVID-19 [31], anticonvulsant, antipyretic [32] and anti-inflammatory [33], as well as the recently reported antihyperlipidemic activity of glycoconjugated phthalimides in mice subjected to a model of dyslipidemia and insulin resistance [34]. This class of compounds has shown pleiotropic effects and stands out for being molecules that can be quickly obtained by organic synthesis, and for allowing modifications and conjugations with other classes of chemical agents. In addition, phthalimides have been shown to be safe molecules with low or no acute toxic effects, which makes them great drug candidates for various therapies [33].

In this sense, the aim of this study was to evaluate, for the first time, the effects of glycoconjugated phthalimides with triazoles in reducing the serum hyperuricemia in hyperuricemic mice induced by potassium oxonate injections, through the analysis of renal function and histology. Subsequently, we evaluated the action of these imidic derivatives in reducing protein degradation in an in vitro model of arthritis and, finally, their therapeutic safety through genotoxicity, mutagenicity, and anti-hemolytic assays.

## 2. Materials and Methods

### 2.1. Synthesis of Glycoconjugated Triazole-Phthalimides

#### 2.1.1. General

All reactions were monitored by TLC analyses containing GF254 and revealed in vanillin. Melting points were determined in an open capillary tube and performed on a PFM II BioSan apparatus. Elemental analyses were carried out on an EA 1110 CHNS-O analyzer from Carlo Erba Instruments. The infrared spectra were recorded on an IFS66 Bruker spectrophotometer using KBr discs. ^1^H and ^13^C NMR were obtained on Varian Unity Plus-300 and Varian UNMRS 400 MHz spectrometers using CDCl_3_ or DMSO-d_6_ as a solvent. The polarimeter used was the Krüss, of 10 cm path length and a concentration of the solution in g/100 mL. The purity of the compounds was determined on an HPLC-DAD Shimadzu Prominence Model.

#### 2.1.2. Typical Procedure to Prepare **F2**, **F3** and **F4**

The *O*-propargyl glucoside (200 mg, 0.69 mmol) was transferred into 50 mL flask, and it was added to 10 mL of dichloromethane. Then, a solution containing 20 mol% copper iodide (0.0268 g, according to alkyne-compound), azido-phthalimides **F1a**, **F1b**, or **F1c** (1.2 equiv), and triethylamine (0.006 g1 drop) was added. The mixture was stirred overnight (12 h) at r.t. (28 °C) under argon atmosphere. Thin layer chromatography (TLC) was used to check the end of the reaction using hexane:EtOAc (7:3) as the developing solvent system. The purification was performed by column chromatography on Merck silica gel 60 (70–230 mesh). After the solvent’s evaporation, the product was crystallized in ethyl acetate.

#### 2.1.3. 4-(4,6-Di-O-acetyl-2,3-dideoxy-α-D-erythro-hex-2-enopyranoside)-O-methyl-1-(2-phthalimidoethyl)-1,2,3-triazole (**F2**)

White solid; 93% yield; mp: 103–106 °C; [α]_D_^25^ + 63.4 (c 1, CH_2_Cl_2_). The ^1^H and ^13^C NMR data were derived from the literature [33,34].

#### 2.1.4. 4-(4,6-Di-O-acetyl-2,3-dideoxy-α-D-erythro-hex-2-enopyranoside)-O-methyl-1-(3-phthalimidopropyl)-1,2,3-triazole (**F3**)

White solid; 80% yield; mp: 125–127 °C; [α]_D_^25^ + 49.6 (c 1, CH_2_Cl_2_). The ^1^H and ^13^C NMR data were derived from the literature [33,34].

#### 2.1.5. 4-(4,6-Di-O-acetyl-2,3-dideoxy-α-D-erythro-hex-2-enopyranoside)-O-methyl-1-(4-phthalimidobutyl)-1,2,3-triazole (**F4**)

White solid; 68% yield; mp: 103–106 °C; [α]_D_^25^ + 37.9 (c 1, CH_2_Cl_2_). The ^1^H and ^13^C NMR data were derived from the literature [33,34].

### 2.2. Model of Hyperuricemia in Mice

**Figure 1:**
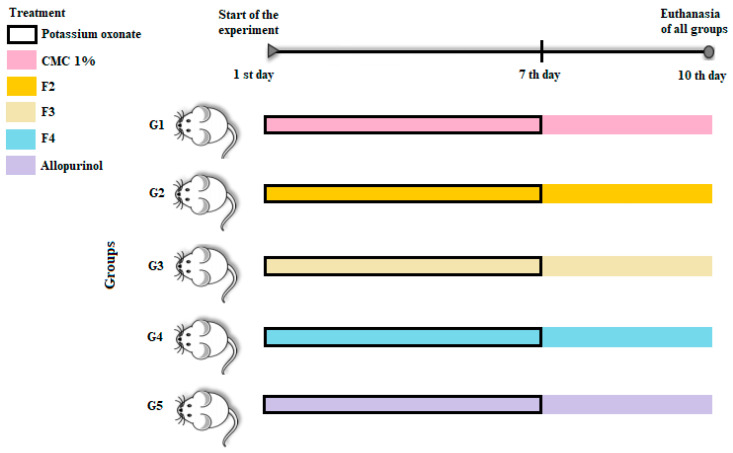
Schematic delineation of the hyperuricemia model. All animals were treated with 250 mg/kg of potassium oxonate for 7 days. G1, negative control and 1% carboxymethylcellulose. G2, **F2** test: treated with 250 mg/kg/day of potassium oxonate, and 20 mg/kg/day **F2**. G3, **F3** test: treated with 250 mg/kg of potassium oxonate and **F3** 20 mg/kg. G4, **F4** test: treated with 250 mg/kg of potassium oxonate and **F4** 20 mg/kg. G5, positive control: treated with 250 mg/kg of potassium oxonate and allopurinol 300 mg/kg. The potassium oxonate, glycoconjugated triazole-phthalimides and 1% CMC vehicle were administered orally for seven days. On the eighth day, potassium oxonate was removed and the animals continued to receive the glycoconjugated phthalimides with triazoles, 1% CMC and allopurinol until the tenth day of research.

In this study, male *Swiss Webster* albino mice aged about eight weeks and having a mean weight ranging from 30 to 35 g were used, provided by the Institute Keizo Asami (iLIKA-UFPE). Throughout the research the animals were kept at constant temperature (23 ± 2 °C) and in a light–dark cycle of 12/12 h. Prior to the induction of the hyperuricemic agent, the animals had free access to standard commercial diet (Labina, Purina Brazil Ltd., São Paulo, Brazil) and water ad libitum. The implementation of this research and its protocols were approved by the Ethics Committee on the Use of Animals (CEUA) of the Federal University of Pernambuco (Proc.: 23076.042305/2017-67), agreeing with the guidelines recommended by the Care and Use of Laboratory Animals (No. 098/2002). Mice were randomly distributed into five experimental groups (G1–G5) with eight (08) animals each, as observed in Figure 1.

### 2.3. Biochemical Analysis

Blood samples were collected from the orbital plexus of animals anesthetized with ketamine and xylazine on the first day of experimentation and the eleventh day, after conclusion of the treatment. The samples were centrifuged at 3000× *g* for 15 min and the serum obtained was separated from the red cells. The levels of uric acid, urea and creatinine were analyzed using laboratory dosage kits by enzymatic reactions (BioSystems^®^, Recife, Brazil) and analyzed by spectroscopy.

### 2.4. Histological Analysis

On the eleventh day of the experiment the animals were sacrificed with the lethal injection of Xilasina anesthetic for removing the kidneys and the subsequent histological analysis. The organs were placed in 10% formalin and later cut, embedded in resin and sliced in a microtome, and the sections were fixed on microscope slides. Staining was done using hematoxylin and eosin (HE).

### 2.5. In Vitro Anti-Arthritic Activity

This method is based on the inhibition of protein denaturation and was evaluated according to Lavanya et al. [35] with modifications, as follows. Samples (50 μL) of 2 and 4 mg/mL **F2**, **F3** and **F4** glycoconjugated phthalimides in 1% CMC solution were added to aliquots (450 μL) of 5% bovine serum albumin (BSA) solution. For 100% denaturation, a negative control group was done by adding distilled water (50 μL) to the same amount of BSA solution. As the standard compound, 2 mg/mL and 4 mg/mL In-domethacin (50 μL) was also added to tubes containing BSA solution for comparison pur-poses. Subsequently, all test tubes were incubated at 37 °C for 20 min, and at 57 °C for 3 min. After the incubation period, 2.5 mL PBS was added to all tubes and the absorbance was read at 416 nm on a spectrophotometer. The activity was calculated according to the formula: % Inhibition = [100 − (Absorbance test − Control product)/Absorbance positive control] × 100.

### 2.6. Assessment of Genotoxicity and Mutagenicity

In total, 20 Swiss Webster albino mice were used to perform the comet and micronuclei assays, and each group consisted of 5 animals (negative control, positive control, **F2**, **F3** and **F4**) maintained in a room at a temperature of 22 °C ± 2 °C and a relative humidity of 50 ± 5%, with a 12 h light/dark cycle. The animals received potable water and feed ad libitum. All experimental procedures with the animals obeyed the norms of the animal ethics committee of the Federal University of Pernambuco. The negative control group received only the 1% carboxymethylcellulose vehicle by gavage at an amount of 1 mL. The positive control group (PC) for the micronucleus received cyclophosphamide (20 mg/kg—Sigma Aldrich^®^, San Luis, MO, USA), a mutagenic agent well established in the scientific literature, by intraperitoneal injection. Meanwhile, for the positive control of the comet assay, slides prepared with animal blood were exposed to 200 mM hydrogen peroxide for 10 min prior to the electrophoresis step of the experiment. The animals treated with phthalimide glycoconjugates received the compounds in a single dose per gavage at the concentration of 20 mg/kg.

For the collection of blood from the animals, they were anesthetized with ketamine (100 mg/kg) and xylazine (10 mg/kg) intraperitoneally and 1 mL of peripheral blood was collected from each animal by retro-orbital puncture. Soon after, the animals underwent euthanasia with a lethal injection of a combination of ketamine (300 mg/kg) + xylazine (30 mg/kg).

The comet assay was performed in a red-light room due to the photosensitivity of the experiment. Initially, 15 μL of blood were homogenized with 100 μL of low melting-point agarose and this mixture was deposited in previously prepared slides, covered with coverslips, and refrigerated at 4 °C for 10 min. Afterwards the slides were placed in lysing solution (2.5 M NaCl, 100 mM EDTA, 10 mM TRIS, 1% Triton X-100, 10% DMSO, pH 10), always under protection from brightness. After 48 h in the lysis solution the slides were electrophoresed with alkaline buffer solution (1 M NaOH and 200 mM EDTA disodium salt, pH 13) for 20 min, with a current of ±300 mA and a potential difference of 32 V. The slides that served as positive control were exposed for 10 min to a 200 mM hydrogen peroxide solution and diluted in the electrophoresis buffer between the steps of lysis and electrophoresis. After this time, the electrophoresis buffer was changed to a new one and the standard protocol of the comet assay was followed. After electrophoresis, the slides were neutralized for 15 min in Tris-HCl 0.4 M buffer, pH 7.5, and fixed for 5 min in absolute alcohol. For development, each slide was stained with 30 μL of ethidium bromide solution (0.0002%, *p*/*v*).

The nucleotides were counted in a fluorescence microscope (Zeiss-Imager, M2, Oberkochen, Germany), with a 40× objective, using the Alexa Fluor 546 filter, counting 100 nucleoids per animal, observing the relationship between tail length and the size of the comet’s head. Each analyzed nucleoid was classified into one of five classes: 0 (no damage); 1 (little apparent damage); 2 (average damage); 3 (medium damage with longer tail); and 4 (maximum damage). Thus, the values obtained for each subject may range from 0 (fully intact: 100 cells × 0) to 400 (maximum damage: 100 cells × 4); to this value we have given the name of damage index (DI) per animal. Thus, the DI was calculated by following the formula:

DI Total = 0 (n° of class comets 0) + 1 (n° of class comets 1) + 2 (n° of class comets 2) + 3 (n° of class comets 3) + 4 (n° of class comets 4).

The frequency of damage (FD%) calculated according to the percentage of all nucleoids with some damage (class 1 through class 4) was also evaluated in relation to the total number of nucleoids counted from class 0 to class 4 (total number) [36], using the formula:

FD = (n° total − n° Class 0) × 100/n° total

In the micronucleus (MN) test, 5 μL of blood were used, which was placed on a slide with acridine orange and covered with a cover slip to evenly spread the biological material. The slides underwent prior preparation to receive the biological material. Initially, they were washed with neutral detergent and distilled water, then bathed in 70% alcohol and deposited in an oven (80 °C) for 15 min. Whilst still being heated, 10 μL of acridine orange (1 mg/mL) was uniformly spread on each slide, then placed to dry at room temperature (minimum 30 min) [37]. Before MN, the cytotoxicity of the treatments was analyzed.

For this, 100 erythrocytes were evaluated, accounting for the proportion of polychromatic erythrocytes (PCE) in relation to total erythrocytes, as follows: PCE/(PCE + NCE), where NCE stands for normochromatic erythrocytes. Thus, the toxicity of each treatment was indicated by a significant reduction (less than 20%) in the percentage of PCE, when the treated group was compared with the NC [38]. For the MN proper, 2000 PCE per animal were analyzed to quantify the presence of micronucleated PCE (PCEMn) [38]. The analysis was performed using a Zeiss-Imager M2 fluorescence microscope, with a 40× objective, using the Alexa Fluor 488 filter.

Statistical analyses for both tests were performed by the Kruskal–Wallis test with a posteriori analysis using the *t*-test strategy paired with Bonferroni correction. In order to verify the efficiency of the tests, the negative control and positive control groups were compared by the Wilcoxon test. The significance level established in all tests was *p* ≤ 0.05 and R software was used for all analyses.

### 2.7. Hemolytic Activity

Hemolytic activity was determined according to Alencar et al. [39], with modifications. Around 4 mL of human blood sample was collected by venipuncture in a sodium citrate tube and then centrifuged at 2500× *g* for 10 min to obtain red blood cells, which were washed three times for 10 min in PBS (0.01 M, pH 7.4), and a 0.5% erythrocytes suspension was made in PBS. An aliquot of this erythrocyte suspension (1.1 mL) was added to 400 μL of serial dilutions of the phthalimide glycoconjugates (1.0 to 0.062 mg/mL), with 0.1% Triton X-100 as the positive control and PBS buffer as the negative control. After 60 min of incubation, it was centrifuged at 2500× *g* for 10 min and the supernatant was read at 540 nm. The test was performed in triplicate and the percentage of hemolysis was determined according to the formula:

$$\%\;\mathrm{of}\;\mathrm{hemolysis}:\;\frac{\mathrm{Abs}\;\mathrm{of}\;\mathrm{treated}\;-\;\mathrm{Abs}\;\mathrm{not}\;\mathrm{treated}}{\mathrm{Abs}\;\mathrm{of}\;\mathrm{Triton}\;X\text{-}100\;-\;\mathrm{Abs}\;\mathrm{not}\;\mathrm{treated}}\times \;100,$$

where Abs is absorbance.

## 3. Results

### 3.1. Chemistry

**Scheme 1:**
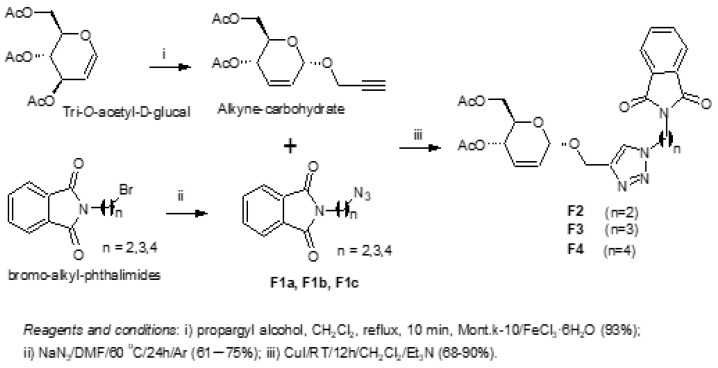
Synthesis of glycoconjugate triazole-phthalimides **F2**, **F3** and **F4**.

Alkyne carbohydrate was first synthesized from the reaction between the tri-*O*-acetyl-d-glucal (Sigma Aldrich^®^) with propargyl alcohol, via a Ferrier rearrangement, in 93% of yields (Scheme 1). In parallel, *N*-azido-alkyl-phthalimides were synthesized from the replacement of bromide with azide ions (NaN_3_) in DMF as a solvent [31]. Then, the Huisgen 1,3-dipolar cycloaddition reaction condition (CuI/Et_3_N/CH_2_Cl_2_) was employed to prepare glycoconjugated triazole-phthalimides **F2**, **F3** and **F4** in good yields (68–90%).

### 3.2. Hyperuricemia in Mice

**Figure 2:**
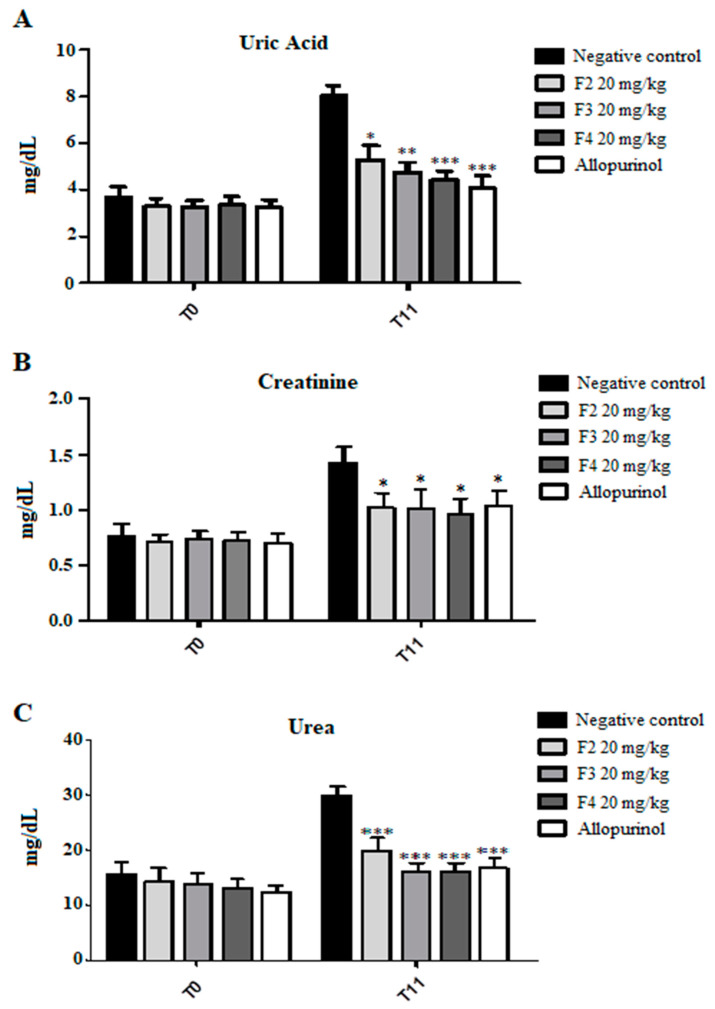
Serum levels of some biochemical parameters from hyperuricemic mice treated with Phthalimides **F2**, **F3**, **F4** and allopurinol. (**A**) Serum levels of uric acid. (**B**) Serum levels of creatinine. (**C**) Serum levels of urea. The statistical differences of the control were determined by multivariate ANOVA followed by the Bonferroni test, * *p* < 0.05, ** *p* < 0.01 and *** *p* < 0.001 vs. negative control.

After a week of administration of potassium oxanate (250 mg/kg), the animals showed elevated serum uric acid (Figure 2A). After treatment with **F2**, **F3** and **F4** (20 mg/kg) and with allopurinol (300 mg/kg) as the positive control, there was a significant reduction (*p* < 0.001) in blood uric acid in the hyperuricemic animals when compared to the negative control group. The negative control group did not present a reduction in serum uric acid. There was no statistically significant difference between the glycoconjugated phthalimides with and without allopurinol.

There was an increase in the serum creatinine level of the animals from the beginning of the experiment to the end (Figure 2B). That rise was more pronounced in the negative control group when compared to **F2**, **F3** and **F4**, and with allopurinol (*p* < 0.001). Again, there was a statistical difference between the imidic compounds and allopurinol. Glycoconjugated triazole-phthalimides also promoted a reduction in the serum urea level of the treated animals compared to the negative control group (*p* < 0.001) (Figure 2C).

### 3.3. Histological Analysis

**Figure 3:**
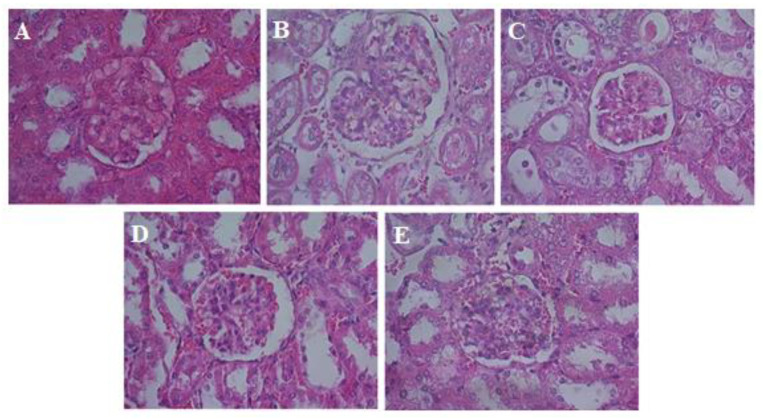
Representative photomicrographs. Kidneys: renal glomeruli and convoluted tubules without morphological changes are visible in the following groups of mice—negative control (**A**), treated with phthalimides **F2** (**B**), **F3** (**C**), **F4** (**D**) and treated with allopurinol (**E**). All images are with 400× magnification.

The histopathological analysis of the kidney demonstrated that hyperuricemia induced by potassium oxonate for ten days did not alter renal morphology. The normal architecture of the kidneys was observed in the control and in treatments with Phthalimides **F2**, **F3** and **F4**; a glomerular architecture could be observed, and the Bowman’s capsule and renal tubules showed no inflammatory changes (Figure 3A–E).

### 3.4. In Vitro Anti-Arthritic Activity

**Figure 4:**
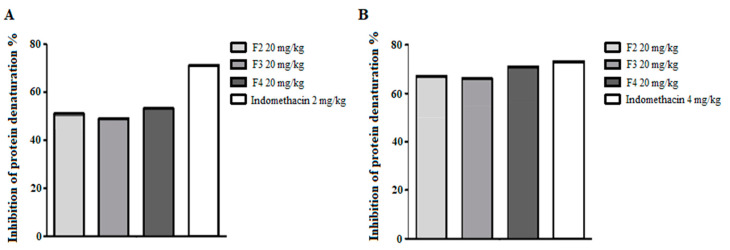
Effect of glycoconjugated triazole-phthalimides on protein denaturation. (**A**) Indomethacin (2 mg/kg). (**B**), 4 mg/kg.

By using the in vitro model of antiarthritis, **F2**, **F3** and **F4** phthalimide derivatives were shown to have potential to inhibit protein denaturation by 51, 49 and 53%, respectively, in comparison to 100% denaturation of the negative control, while the positive control indomethacin at the concentration of 2 mg/kg reduced protein denaturation by 71% (Figure 4A). Doubling the concentration of indomethacin, the reduction in protein denaturation (73%) was similar to the previous, but the effects of **F2**, **F3** and **F4** were better, reducing protein denaturation by 67, 66 and 71%, respectively (Figure 4B).

### 3.5. Assessment of Genotoxicity and Mutagenicity

**Figure 5:**
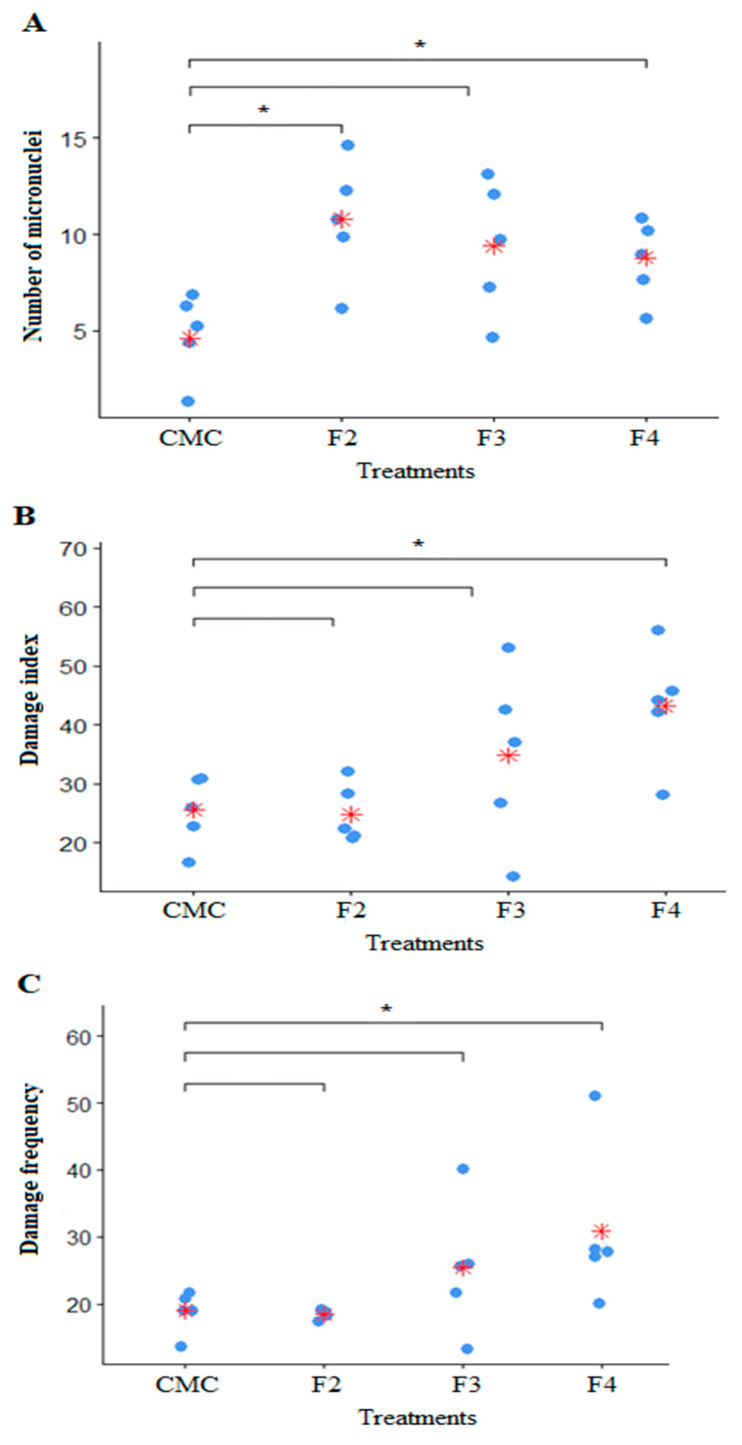
Comet essay. (**A**) micronucleus test. (**B**) Comet test to evaluate damage index and (**C**) comet test to evaluate damage frequency. The statistical differences of the control were determined by Wilcoxon’s non-parametric test. * *p* < 0.05 vs. negative control (red asterisk means the non-parametric mean of each group).

Glycoconjugate phthalimides with triazoles **F2** and **F4** had a higher number of micronuclei than the group treated with 1% CMC control, when compared by the Wilcoxon nonparametric test (*p* < 0.05 significant difference). Compound **F3**, despite having a higher micronucleus number than the 1% CMC control, showed no statistically significant difference (Figure 5A). In the evaluation of the damage index (DI), only compound **F4** presented a statistically significant difference when compared to the negative control group (*p* < 0.05). The glycoconjugates of two- and three-carbon phthalimides in the aliphatic chain (**F2** and **F3**) did not present a statistically significant difference when compared to the 1% CMC group (Figure 5B). For the frequency of damage (FD), the phthalimide glycoconjugate with triazol **F4** presented a statistically significant difference in comparison with the 1% CMC negative control group (*p* < 0.05). Compounds **F2** and **F3** did not present statistical differences compared to the negative control group (Figure 5C).

### 3.6. Hemolytic Activity

**Figure 6:**
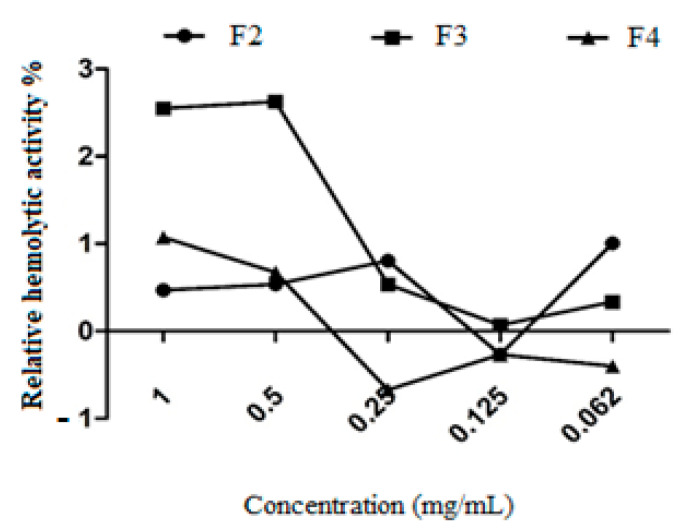
Percentage of hemolysis caused by glycoconjugated triazole-phthalimides.

Phthalimide glycoconjugates with triazole had a low percentage of erythrocyte hemolysis. The highest percentage with the highest concentration of compounds was compound **F3**, which presented 2.2% hemolysis. Compounds **F2** and **F4** had hemolysis percentages below 2% (Figure 6).

## 4. Discussion

Alkyne carbohydrate was first synthesized from the reaction between the tri-*O*-acetyl-d-glucal (Sigma Aldrich^®^) and propargyl alcohol, via a Ferrier rearrangement, in 93% of yields (Scheme 1) [40]. In parallel, *N*-azido-alkyl-phthalimides were synthesized from the replacement of bromide with azide ion (NaN_3_) in DMF as a solvent [33]. Then, the Huisgen 1,3-dipolar cycloaddition reaction condition (CuI/Et_3_N/CH_2_Cl_2_) was employed to prepare glycoconjugated triazole-phthalimides **F2**, **F3** and **F4** in good yields (68–90%). The molecular structure of the phthalimide syntheses was confirmed by IR, ^13^C NMR, and ^1^H NMR by the similarity of the spectra reported by Assis et al. [33] and Silva Júnior et al. [34].

Excess serum uric acid, called hyperuricemia, is a risk factor for the development of various diseases such as gouty arthritis, diabetes, cardiovascular diseases, metabolic syndrome, hypertension, and kidney disease [41]. Due to this range of harmful effects to human health, excess uric acid in the blood should be treated in order to avoid the development of serious health problems. In this study, an inhibitor of uricase, potassium oxonate, at the dose of 250 mg/kg was used. This enzyme enables the oxidation of uric acid into 5-hydroxysucrate, a more soluble compound, which is excreted by the renal emollient route. This compound, when given for seven days, increased the serum levels of uric acid, creatinine and urea. Such elevations in these metabolites by the administration of potassium oxonate has already been demonstrated in other studies, due to a decrease in the activity of basolateral membrane transporters of the kidneys, as demonstrated in rat experiments, causing a reduction in the expressions of the renal transporters rOAT1, rROAT3 and ROCT2, evaluated by mARN and expressed protein analysis [42,43].

Hyperuricemia can lead to changes in the metabolism of lipids and carbohydrates. Triglyceride biosynthesis can occur in situations of excess fructose, using aldolase B (Aldo B) and fatty acid synthase (FAS) enzymes. However, when there is a significant increase in uric acid due to nucleotide turnover, resulting from hyperproteic diets, this can lead to a state of mitochondrial oxidative stress (mtROS), and this state reduces the activity of the enzyme aconitase (ACO2) in the Krebs cycle. Because of this reduction, there is an increase in the substrate of ACO2, which is citrate. This citrate leaves the mitochondria towards the cytosol, where it accumulates and becomes a substrate for the formation of triglycerides, through the action of the enzymes’ fatty acid synthase and ATP citrate lyase (ACL) [44,45,46,47]. In addition to this mechanism of triglyceride synthesis using excess carbohydrate, hyperuricemia may lead to a decrease in the activity of the enzyme enoyl CoA hydratase 1, a key enzyme in the beta-oxidation process of fatty acids. This leads to the accumulation of lipids in liver tissue, leading to steatosis and nonalcoholic fatty liver disease (NAFLD) [44,45].

Studies using a model of hyperuricemia with induction by potassium oxonate demonstrated a relationship between the increase in uric acid and insulin resistance; therefore, hyperuricemia is a possible factor triggering diabetes [48]. This process is due to dysfunctions in the insulin receptors that are fundamental for capturing glucose in the cell. Some of the signals from insulin receptors are transmitted through pathways that surround the insulin substrate receptor (IRS), and their phosphorylation leads to inactivation. A state of hyperuricemia may lead to a reduction in IRS1 activity, thus causing a state of insulin resistance [48]. Uric acid is also able to reduce the vasodilator potency of insulin, which is critical for the process of glucose delivery in the skeletal muscles [49]. This inactivation may therefore allow excess uric acid to cause diabetes mellitus, which has been strongly correlated in epidemiological studies with humans [50,51,52,53].

Because of the large number of comorbidities that excess uric acid can cause, such as lipid and carbohydrate changes, which are reflected in chronic diseases, there is a search for compounds that can reduce hyperuricemia and improve the consequences of the increase in this metabolite, such as the glycoconjugated triazole-phthalimides **F2**, **F3** and **F4** presented in this study. Phthalimide derivatives have already shown the effects of reducing serum uric acid, emerging as inhibitors of xanthine dehydrogenase and xanthine oxidation, key enzymes in uric acid biosynthesis [54].

Although other studies using potassium oxate as an inducer of hyperuricemia have demonstrated that this increase in uric acid was able to cause alterations in renal histology [42,43,55], in this study no alterations were observed in kidney morphology, presenting normal structures such as Bowman’s capsule, glomeruli and proximal tubules in all groups (Figure 6). This type of morphological alteration may have been observed in other studies because in these other studies there were, in addition to potassium oxonate, other agents inducing hyperuricemia, such as yeast and 2.5% uric acid solution in the experimental animals, which provoked, besides the morphostructural changes in the kidneys, an intense cellular infiltrate characteristic of the inflammatory process [42,43,55].

The increase in serum uric acid is a fundamental condition for the development of gout, a metabolic disease that most frequently affects middle-aged men, the elderly, and women in the postmenopausal period, resulting from the accumulation of mainly monosodium urate (UH) crystals in the joints, skin, bones, and synovial fluid of the carriers. These crystals of monosodium urate are solid forms of uric acid, which is the end product of the metabolism of nitrogenous compounds [56,57]. Serum uric acid levels can increase in three ways: when there is an increase in production due to a higher protein degradation with generation of many nitrogenous metabolites, when there is little excretion due to some renal dysfunction, or when there is the pharmacological interference of some therapeutic agent [56]. The inhibition of protein denaturation is therefore a tool to prevent the installation and progression of the inflammatory reaction of gout. In these studies, the glycoconjugated phthalimides with **F2**, **F3** and **F4** triazoles presented high percentages of inhibition of protein denaturation in vitro, with values very close to indomethacin, a non-steroidal anti-inflammatory drug (NSAID), which acts non-selectively on cyclooxygenase (COX-1 and COX-2) and which, because of this non-selectivity, can cause problems in the gastrointestinal tract, especially with prolonged use [58]. Inhibition was shown to be dose dependent, with a higher inhibition percentage at the dose of 4 mg/kg as compared to the 2 mg/kg dose.

The pharmacological agents used in the most diverse therapies can cause cellular damage by inducing defects in DNA replication and even in gene expression, generating malformed proteins that can lead to pathological processes such as cancer and cell death. These harmful effects on the genetic material are caused by genotoxic compounds, and it is therefore of great importance that in the search for new molecules with therapeutic purpose, genotoxicity is evaluated [59]. In this study, the glycoconjugated phthalimides **F2** and **F4** presented in the micronucleus test a number a little larger than that of the 1% CMC negative control, which did not occur with the phthalimide glycoconjugate **F3**. This small increase in the number of micronuclei in the compounds **F2** and **F4** can be considered a small effect, since the dosage used was 20 mg/kg, a dose much higher than that of another study using Tebuconazole at 300 μg/L, a triazole used as fungicide [60]. In the evaluation of the damage index and damage frequency by the comet assay, only compound **F4** presented a significant difference when compared with the 1% CMC control. However, despite this small change in the comet assay, cyclic imides such as thalidomide had their genotoxic effects evaluated, and did not present themselves as potent agents that could generate damage to the genetic material. This was corroborated by these results, demonstrating that the use of a dosage course is also necessary to evaluate at what point damaging effects to the DNA are initiated, thus conferring greater therapeutic safety to the compounds [61].

In the evaluation of the hemolytic capacity of the glycoconjugated triazole-phthalimides, serial doses of 1 to 0.062 mg/mL were used. The percentage of hemolysis did not exceed 3%, indicating the safety of these compounds in terms of the integrity of the erythrocyte membrane structure. This result corroborates another study using phthalimides and evaluating their capacity for hemolysis, also finding low rates [62].

Glycoconjugated triazole-phthalimides had a very satisfactory antihyperuricemic effect in the model of induction of hyperuricemia by potassium oxonate, reducing levels of serum uric acid, creatinine and urea. The pharmacological action found in this study is a result of great importance due to the diverse biological complications that the excess of uric acid can cause in the human organism. The elevation of serum uric acid may compromise the normal metabolic pathways of carbohydrates and lipids [63], which may lead to situations of insulin resistance and dyslipidemia [64] due to excess triglycerides [65], for example, and therefore reducing uric acid levels is very important to avoid the progression of metabolic diseases [66].

The chemical molecules tested in this study were also effective in inhibiting protein denaturation in vitro. This potential for the inhibition of protein degradation indicates a pharmacological effect against the development of gouty arthritis, a metabolic disorder originating from hyperuricemia, which arises from excess purine degradation. Avoiding the development of gouty arthritis is of paramount importance, since the disease leads to a decrease in the activity of the patient, mainly due to excessive pain, and these are among the most frequent causes of disability [67,68]. This ability to inhibit protein denaturation of glycoconjugated phthalimides is in addition to their antihyperuricemic effect. Therefore, these molecules are capable not only of upregulating elevated uric acid levels in the blood, but also of inhibiting the formation pathway of this nitrogen product by inhibiting protein degradation, and thus avoiding the formation of gouty arthritis and of metabolic disorders.

Phthalimide compounds **F2**, **F3** and **F4** showed satisfactory therapeutic safety. In the genotoxicity test of the micronucleus the compounds **F2** and **F4** showed a small increase in the micronucleus number. In the comet test, only compound **F4** presented a higher damage index and a higher frequency of damage, but this was very close to the value of the control group. These data indicate that compounds **F2**, **F3** and **F4** have few effects on the genetic material, and that comparative dose studies are required to determine at which concentration of these compounds an increase in the number of micronuclei and damage in the comet assay begins to occur. Glycoconjugated triazole-phthalimides have been tested for their ability to induce hemolysis, and the results suggest that they are safe substances and have low erythrocyte hemolysis percentages, further reinforcing the safety of the use of glycoconjugated triazole-phthalimides and the minor risk of hemorrhage involvement and clinical severities in potential therapeutic applications [69].

The results of this study indicate a promising pharmacological action of triazole-phthalimides in reducing serum levels of uric acid and inhibiting protein denaturation in a hyperuricemic and anti-arthritic model. These effects are encouraging because of the immense range of actions deleterious to the body that excess uric acid and nitrogenous excreta can cause. Metabolic and systemic changes can lead to the inactivation and even death of patients. Therapeutic safety tests have shown that the phthalimides tested do not have any strong effects on the models tested. Despite these results, further studies are needed to elucidate the mechanisms of action of these molecules, as well as their pharmacokinetics, so that they may become in the future a therapeutic agent against hyperuricemia and its complications, such as gouty arthritis.

## 5. Conclusions

Taken together, all the results indicate that glycoconjugated triazole-phthalimides have an action against hyperuricemia and its secondary complications such as gouty arthritis, making these molecules strong candidates for therapeutic agents for excess uric acid. Although the new glycoconjugate triazole-phthalimides are interesting drugs due to their potential anti-hyperuricemic actions, further studies are important in order to understand the mechanism of action.

## Data Availability

The data presented in this study are available on request from the corresponding author.
